# Are AI weather models learning atmospheric physics? A sensitivity analysis of cyclone Xynthia

**DOI:** 10.1038/s41612-025-00949-6

**Published:** 2025-03-07

**Authors:** Jorge Baño-Medina, Agniv Sengupta, James D. Doyle, Carolyn A. Reynolds, Duncan Watson-Parris, Luca Delle Monache

**Affiliations:** 1 Center for Western Weather and Water Extremes, Scripps Institution of Oceanography, University of California San Diego, San Diego, CA USA; 2 U.S. Naval Research Laboratory, Monterey, CA USA; 3 Halıcıoğlu Data Science Institute, University of California San Diego, La Jolla, CA USA

**Keywords:** Atmospheric dynamics, Statistics

## Abstract

Artificial Intelligence (AI) weather models are explored for initial condition sensitivity studies to analyze the physicality of the relationships learned. Gradients (or sensitivities) of the target metric of interest are computed with respect to the variable fields at initial time by means of the backpropagation algorithm, which does not assume linear perturbation growth. Here, sensitivities from an AI model at 36-h lead time were compared to those produced by an adjoint of a dynamical model for an extreme weather event, cyclone Xynthia, presenting very similar structures and with the evolved perturbations leading to similar impacts. This demonstrates the ability of the AI weather model to learn physically meaningful spatio-temporal links between atmospheric processes. These findings should enable researchers to conduct initial condition studies in minutes, potentially at lead times into the non-linear regime (typically >5 days), with important applications in observing network design and the study of atmospheric dynamics.

## Introduction

A significant portion of atmospheric predictability is determined by the initial condition uncertainty. Small errors in the initial condition will grow as a function of forecast lead time amplified by the nonlinear and chaotic nature of the atmosphere^1^. Sensitivity analyses allow one to examine how a target forecast metric/variable of interest (K), e.g., precipitation over the Western US, responds to (infinitesimal) perturbations to the atmospheric patterns at the initial time (Xi) at an upstream location, i.e., the gradient ∂K/∂Xi. Sensitivity studies are useful for several applications, such as targeted observing^2^, observing network design^3^, parameter estimation^4^, and data assimilation^5^. The sensitivities are a representation of the links between the atmospheric variables through space and time, and can be potentially used to understand the physical processes that impact the forecast evolution. The latter is explored here with the new generation of Artificial Intelligence (AI) data-driven weather models to examine the physicality of the relationship learned.

AI data-driven models are revolutionizing the field of weather forecasting, showing competitive forecast skill with state-of-the-art dynamical (i.e., physics-based) models^6–12^, at a much lower real-time computational cost (after training). They are based on neural networks^13^, which are combinations of differentiable linear operators and nonlinear activation functions, optimized by means of the backpropagation algorithm^14^, which modern libraries support via auto-differentiation. The backpropagation algorithm, along with its associated libraries, enables the computation of gradients (or sensitivity fields) by applying the “chain rule”. This process backpropagates information from the output layer (target metric of interest) to the input layer (initial condition). Remarkably, this can be done in a matter of minutes using a single CPU, or even seconds with a GPU.

The aforementioned properties make AI models attractive tools for initial condition sensitivity studies^15^, and can overcome current limitations of dynamical-based methods, which typically rely on the adjoint of a linearized version of the model, which linearizes the evolution of perturbations along the initial trajectory^16^. This generally implies that this method cannot be used as a sensitivity tool for lead times larger than a few days, beyond which the assumption of linearity typically does not hold. Moreover, the formulation of the adjoint is not trivial, especially with process-based parameterizations, and requires running the non-linear dynamical model, increasing the computational burden of the methodology. In turn, sensitivities from AI data-driven models are not constrained by linearity assumptions and are simpler to compute. For instance, recently the predictability limit of the 2021 Pacific Northwest heatwave was efficiently explored using backpropagation to analyze forecast error at weather and sub-seasonal timescales^15^.

Here, the ability of an AI data-driven model to replicate the sensitivities of cyclone Xynthia is examined, using values from the adjoint of a physics-based model^17^ as reference. This study will help elucidate whether these models are using information from physically-realistic relationships to produce the forecasts, and therefore potentially be used as initial condition sensitivity tools.

## Results

### Case study: cyclone Xynthia

Strong winds and heavy precipitation associated with mid-latitude cyclones can have significant socio-economic impacts. As a result, understanding the dynamics behind these events and enhancing forecast accuracy are crucial for society. Moreover, climate change simulations project an increase in both the intensity and frequency of precipitation extremes linked to extratropical cyclones under future emission scenarios, compared to pre-industrial conditions^18^. Cyclones are complex phenomena, often driven by non-linear interactions between dynamic and thermodynamic processes^19–24^. In this context, cyclone Xynthia provides an ideal case study to explore the potential of AI-driven models as tools for sensitivity analysis and to evaluate the physical realism of the relationships these models uncover.

**Fig. 1:**
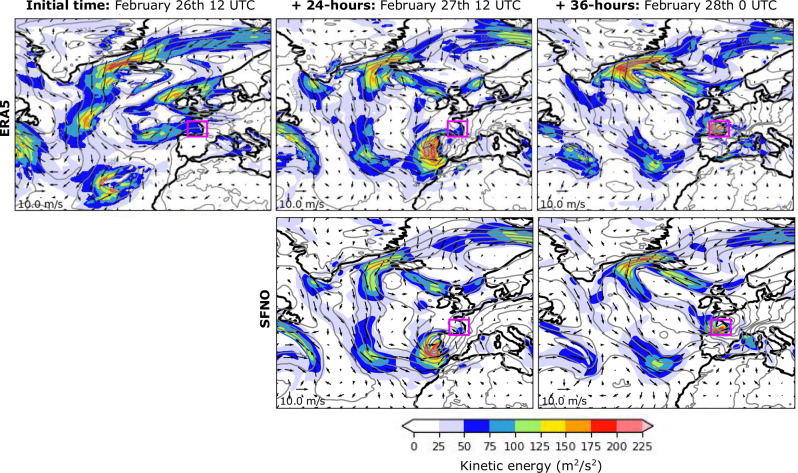
Evolution of cyclone Xynthia. First row: Evolution of cyclone Xynthia from February 26th at 12 UTC to February 28th at 00 UTC in the ERA5 reanalysis. Second row :SFNO predictions of cyclone Xynthia for 24 and 36 h ahead. Colors represent values for the kinetic energy, contours for the mean sea level pressure (from 972 to 1036 every 4 hPa), and vectors for the intensity of surface wind (every 4°, i.e., 16 grid-boxes). The magenta box represents the area used to compute the sensitivities.

**Fig. 2:**
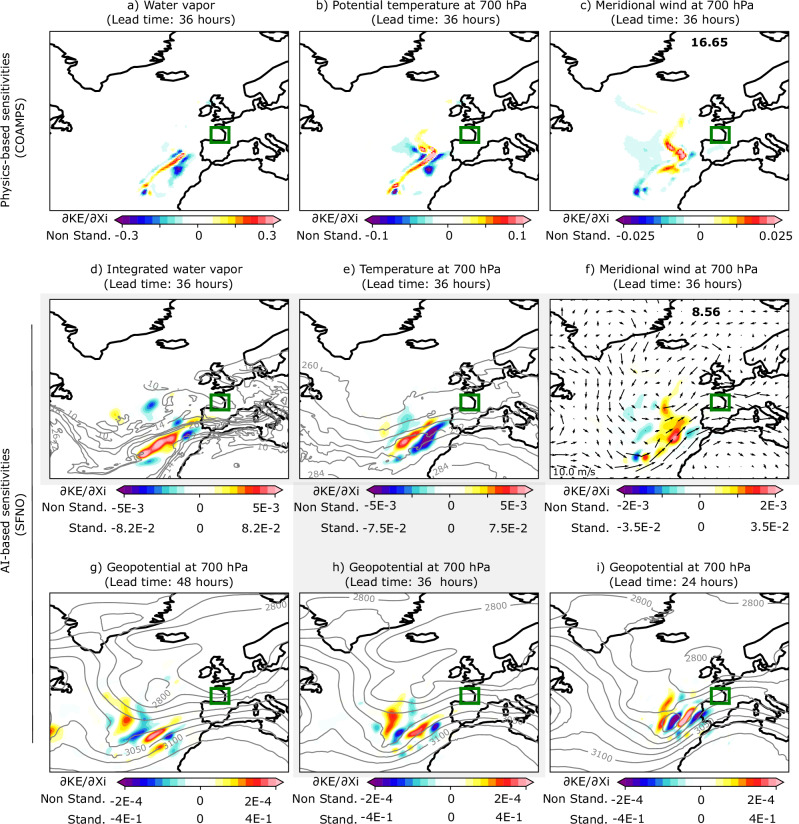
Comparison of physics-based and AI-based sensitivity fields for cyclone Xynthia. Physics-based sensitivity fields of the kinetic energy over the Bay of Biscay (green box) at 36 h of lead time for **a** water vapor (g/kg), **b** 700-hPa potential temperature (m^2^ s^−2^ K^−1^), and **c** 700-hPa meridional wind (m/s), as computed in D14. AI-based sensitivities of the kinetic energy for **d** integrated water vapor (kg/m^2^), **e** 700-hPa air temperature (K), and **f** 700-hPa meridional wind (m/s). The bottom row shows the 700-hPa geopotential (m^2^/s^2^) sensitivity fields at **g**) 48 h, **h** 36 h, and **i** 24 h of lead time. Contours in **d**), show the total column water vapor from 10 to 30 every 4 kg/m^2^ at initial time, February 26th 12 UTC. Similarly, in **e**) the contours show the values of temperature every 4K, while in (**g**–**i**), the 700-hPa geopotential is displayed every 50 (m^2^/s^2^) from 2800 to 3200. Vectors are used to represent the wind intensity at 700 hPa in **f**). Gray shading indicates the panels representing the AI-based sensitivities at 36 h of forecast lead time. The sensitivities are represented with color bars with different scales to visualize the spatial structures found across variables. The standardized scales are also shown for comparison among AI sensitivities.

Cyclone Xynthia was an impactful event that caused important socio-economic damages in Western Europe, leading to high winds, extreme precipitation, flooding, and large ocean waves, resulting in more than 50 deaths across several European countries. It originated on February 26th, over a low-pressure center situated south of the Azores Islands (996 hPa, see Fig. 1), on the edge of an atmospheric river (AR). ARs are elongated structures in the atmosphere transporting moisture from the tropics to the mid-latitudes, and are often associated with extreme precipitation^2^. In particular, this AR had a length of ~6500 km in the southwest-to-northeast direction—from the Azores to Southern Iberia—with an averaged width of ~700 km, and carried up to ~30 kg of water vapor per meter square (Fig. 2d). The system rapidly intensified as it passed over the coast of Portugal and Northern Spain, reaching Western France on February 28th with winds greater than 30 m/s, 7.5 m waves, and producing almost 2 m of rain. After March 1st, the intensity of the event decreased as it moved inland towards Central Europe. The evolution of cyclone Xynthia is shown in Fig. 1, and the reader is referred to the literature for more details^25^.

A sensitivity analysis of cyclone Xynthia using a dynamical and adjoint model was performed in a reference study^17^ (hereafter referred to as D14) to investigate the drivers and predictability of this extreme event. In the latter study, sensitivity fields of kinetic energy (KE) over the Bay of Biscay (highlighted by the magenta box in Fig. 1) were computed for a 36-h forecast, using February 26th at 12 UTC as the initial condition. These fields were obtained through the Coupled Ocean-Atmosphere Mesoscale Prediction System (COAMPS) adjoint model^26^, which employs a non-hydrostatic dynamical core. The model was resolved over a nested domain with 45-km horizontal grid spacing for the coarse mesh and 15-km grid spacing and included a microphysics parameterization (predicting cloud water, rainwater, snow, and graupel concentrations) and a subgrid-scale parameterization for deep convection^27^. The sensitivities from D14 are replotted here to facilitate comparison with the AI-based results.

**Fig. 3:**
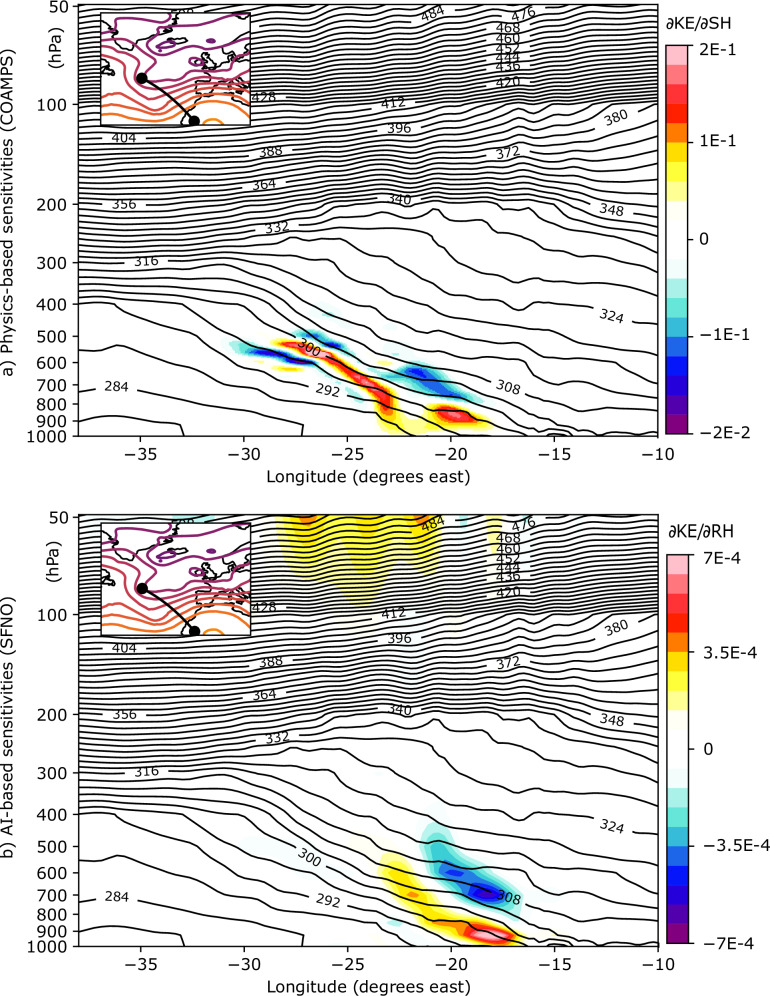
Vertical cross-section of the sensitivity fields of kinetic energy over the Bay of Biscay for cyclone Xynthia. **a** Vertical cross-section of the physics-based sensitivities of Kinetic Energy (KE) relative to the Specific Humidity (SH, g/kg) at 36 h of forecast lead time. The inner plot shows the geopotential at 500 hPa every 100 hPa, and the exact location of the cross-section (solid black line). Contours display ERA5’s potential temperature every 4 K. **b** Vertical cross-section of the AI-based sensitivities of Kinetic Energy (KE) relative to the Relative Humidity (RH, %) at 36 h of forecast lead time.

The comparison presented is purely qualitative due to significant differences in how sensitivities are computed between the AI and D14 approaches. First, the AI model is initialized using the European Centre for Medium-Range Weather Forecast Reanalysis version 5 (ERA5^28^) data, with initial condition sensitivities computed relative to this dataset, whereas the COAMPS model is initialized from the National Oceanic and Atmospheric Administration (NOAA) Global Forecast System (GFS). Second, the set of variables simulated differs between the models. Therefore, AI-based sensitivities are compared with the closest available proxies in COAMPS. For example, relative humidity from the AI model is compared to specific humidity in COAMPS, as shown in Fig. 3. Third, the vertical and spatial resolutions also vary. While COAMPS uses z-sigma vertical levels and a grid mesh of 45 km (coarse mesh) or 15 km (fine mesh) horizontal grid increments, the AI model simulates variables at 13 pressure levels on a 0.25° latitude-longitude grid. Fourth, the method for calculating kinetic energy differs slightly: D14 includes the vertical wind component, whereas the AI model considers only the zonal and meridional components (see section Methods). All of these factors should be taken into account when analyzing the results in the following sections.

Additionally, cyclone Xynthia was included in the AI model’s training dataset (see section Data), but the model was not specifically optimized for 36-h forecasts during training (see section Methods), which is the lead time considered in this study. Even if specifically optimized to this aim this would not pose a concern, since the primary goal is to validate the spatio-temporal relationships the model has learned (and not measuring forecasting error), framing these kinds of experiments in the training dataset is a well-established practice in the literature^29–31^. Moreover, AI models typically minimize an error function without incorporating explicit physical knowledge in their design. Cyclone Xynthia appeared in only 0.01% of the training samples and covered 0.06% of the global grid. This was a rare event, following an unusual trajectory for cyclones in that region^25^, which typically move easterly toward southern Europe when originating near the Azores^32^. Given these factors, the contribution of Xynthia to the overall model loss and coefficient optimization is likely negligible. Therefore, Xynthia serves as a valid case study for analyzing spatio-temporal dependencies without concerns about overfitting. In fact, the demonstration of coherent spatio-temporal links would provide evidence of generalization, while the emergence of a non-physical pattern would suggest overfitting.

### Forecasting cyclone Xynthia

Figure 1 shows the evolution of cyclone Xynthia as represented by ERA5^28^ (first row), which is used as ground truth in this study, and as predicted by the Spherical Fourier Neural Operator (SFNO^7^) AI model (second row; see details in “Methods” section). The SFNO predicts a rapid intensification of Xynthia with winds carrying kinetic energy approaching 200 m^2^/s^2^ and the low-pressure system moving towards the coast of Portugal by February 27th at 12 UTC, which is consistent with ERA5. By February 28th at 00 UTC, Xynthia reached the Bay of Biscay and Western coast of France. The SFNO accurately captures the strong pressure gradient in this area and predicts slightly less kinetic energy than ERA5, but still surpassing values of 200 m^2^/s^2^ in the region. A comparison of the kinetic energy at the 36-h forecast time between SFNO and the Integrated Forecasting System (IFS),—ECMWF’s state-of-the-art dynamical prediction model—shows similar values of root mean squared error between models, with 58, and 82 m^2^/s^2^, respectively. These values were averaged over the Bay of Biscay on February 28th at 00 UTC (magenta box in Fig. 1), and ERA5 was used as the ground truth. The IFS forecast was initialized using its own analysis dataset, which may partly explain the slightly higher RMSE observed in IFS compared to SFNO when validated against ERA5.

### Initial condition sensitivities

Figure 2 shows the sensitivity fields of kinetic energy (KE) over the Bay of Biscay (highlighted by the green box in Fig. 2; see the “Methods” for further details) for a forecast lead time of 36 h, with the initial condition set to 12 UTC on February 26th. Specifically, the 45-km physics-based sensitivities for water vapor (g/kg), 700-hPa potential temperature (m^2^ s^−2^ K^−1^), and 700-hPa meridional wind (m/s) from D14 are compared to the AI-based sensitivities for integrated water vapor (kg/m^2^), 700-hPa air temperature (K), and 700-hPa meridional wind (m/s), respectively. The 700 hPa level is chosen because it exhibits prominent behavior during the Xynthia cyclone (see Fig. 3). To improve the visualization, the color scales for each variable are different. For the AI-based maps, there are two color scales: one for the sensitivities computed relative to the standardized version of the inputs (for direct comparison with the physics-based sensitivities) and one for the sensitivities computed relative to the non-standardized version of the inputs (which allow comparison among AI-sensitivities, indicating the relative importance of each variable to the KE; see Methods section). Positive values (shown in red) indicate areas where infinitesimal increases in the initial condition fields lead to an increase in KE at the final time, while negative values (shown in blue) indicate areas where infinitesimal decreases in the initial conditions lead to an increase in KE.

The results reveal that a small moisture filament, ~40 km wide, within the atmospheric river (AR) is the main contributor to cyclone development (Fig. 2a, d). This filament extends west of the Azores, in a southwest-northeast direction, toward the coast of Portugal, with maximum sensitivity values between the 500 and 1000 hPa levels (Fig. 3a). Temperature sensitivities align with this moisture filament (Fig. 2b), while wind sensitivities are concentrated in the short-wave troughs over the Central and North Atlantic. This suggests that stronger southerly winds at the initial time lead to increased kinetic energy at the final time (Fig. 2c). Additionally, negative water vapor sensitivities flank the positive moisture filament within the AR, indicating that an intensification of the winds results from increasing the moisture gradient in this region (Fig. 2a). The AI-based and physics-based sensitivities are remarkably similar, showing nearly identical spatial structures for the variables examined. For instance, for the meridional wind, the S-shaped sensitivity structure west of the coast of Portugal along the short-wave trough (Fig. 2c) is well captured by the AI model (Fig. 2f). Both the integrated water vapor and temperature fields also show elongated structures along the AR. However, while the AI-based model’s maximum sensitivity values are generally lower than those of the physics-based model, as clearly illustrated in the meridional wind panels (Fig. 2c, f), the aggregated sensitivities across the map (shown in the top-right values) are of similar magnitude. This can be explained by the non-zero influence of non-relevant features due to complex interactions within the neural network. Also, despite having a higher-resolution grid, the AI-based model displays coarser sensitivities than the physics-based ones. This is likely due to the effective resolution of AI models, which is documented to be lower than the resolution used during the model’s training^33^.

The AI-based sensitivities for geopotential are also shown (Fig. 2g–i), and they are notably more pronounced than those for the other variables (see standardized scales). These sensitivities exhibit wave-like patterns, which, based on experience from adjoint sensitivity studies^34^, may result from the projection of sensitivities onto a Rossby wave packet in the waveguide. This packet interacts with precipitation and diabatic processes, propagating downstream. As the lead time increases from 24 to 48 h, these wave-like patterns shift westward, moving further offshore and upstream of the response function. This westward shift is consistent with the expected eastward propagation of the signal over time, driven by the background westerly winds, and highlights the physical consistency of the relationships learned by the AI model.

Figure 3b shows the relative humidity (RH) sensitivities for a vertical cross-section connecting two locations in the northwest-southeast direction (see the inner panel in Fig. 3), crossing the atmospheric river (AR). The positions of these two locations were determined based on the sensitivity maximums shown in Fig. 2. These same two locations are used to plot the specific humidity (SH) sensitivities from the physics-based model in D14 (Fig. 3a). As in Fig. 2, the comparison between the approaches is qualitative, since SFNO (COAMPS) does not include SH (or RH) in its variable set. The x-axis represents the longitudes between the two locations, and the y-axis corresponds to pressure levels (refer to section Data for a complete list of pressure levels in SFNO). The areas of maximum sensitivity are located between longitudes −25° and −15° spanning from the surface to 450 hPa. The positive-negative dipole indicates that an increased moisture gradient within the AR at the initial time leads to a corresponding increase in kinetic energy over the Bay of Biscay at the final time. This dipole tilts against the sloping warm frontal zone. Overall, the vertical sensitivities align well with the physics-based fields, with only minor differences in the positions of the maximum values. These differences can be partially explained by the fact that the two models are fundamentally different, with distinct horizontal and vertical resolutions, as well as notable methodological differences in how predictions are generated.

Finally, a positive sensitivity is observed at the upper levels of the atmosphere, which may suggest a spurious or noisy correlation learned by the AI model. This is particularly noteworthy, since to our knowledge there is no physical principle supporting such a relationship between upper-level atmospheric conditions and low-level final-time kinetic energy. Identifying these non-physical links is important in AI-based weather modeling, as it could help guide the development of more reliable and physically consistent models in the future.

### Evolution of sensitivity-based perturbations

**Fig. 4:**
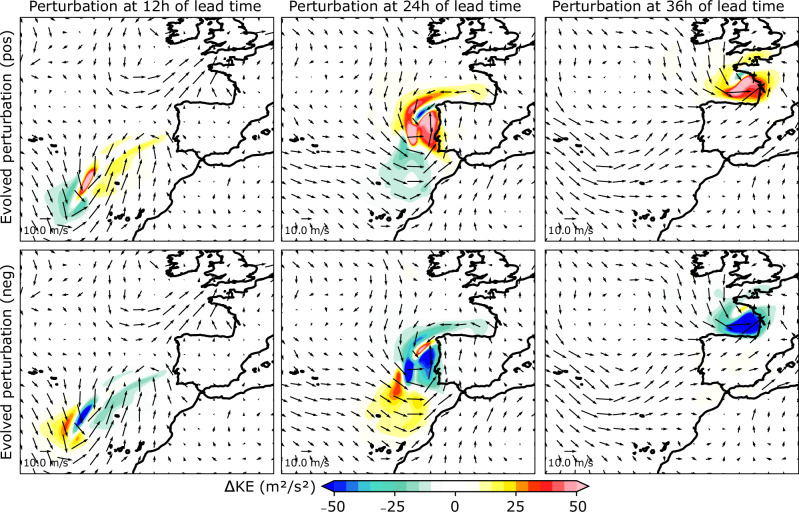
Evolved perturbation fields for the kinetic energy at 12, 24 and 36 h of lead time. First row: Evolved perturbation fields for the kinetic energy (KE) at (from left to right) 12, 24 and 36 h of lead time, where the sensitivity-based perturbations were added to the initial condition (positive). Second row: Evolved perturbation fields for the kinetic energy (KE) at (from left to right) 12, 24 and 36 h of lead time, where the sensitivity-based perturbations were removed from the initial condition (negative). Surface wind intensity from ERA5 at each time is represented by vectors.

Sensitivity-based perturbations, scaled to align with estimates of initial condition uncertainty (see Methods), are applied to the variables at the initial time. Figure 4 shows the difference between the perturbed and the control forecasts for the kinetic energy at 12, 24, and 36 h of forecast lead time. Two different perturbed forecasts are generated. The first one adds the sensitivity-based perturbation to the initial condition (positive perturbation, first row), while the second one changes the sign (negative perturbation, second row). The evolved kinetic energy perturbations grow in intensity from overall 5–25 m^2^/s^2^ at 12 h of forecast lead time to 15–50 m^2^/s^2^ at final time, and they follow the trajectory of the low-pressure system at each forecast lead time until it reaches the Bay of Biscay on February 28th at 00 UTC. The positive perturbations, which added moisture to the initial condition and increased the moisture and temperature gradients along the AR, simulate an intensification of the winds related to Xynthia. In contrast, negative perturbations produce almost the opposite response, overall decreasing the kinetic energy of Xynthia relative to the control forecast. This is somewhat expected since perturbation fields were built based on the sensitivity fields. These properties were also identified in D14 (see Figure 12 therein). The symmetry between the positive and negative perturbations at final time is evidence of quasi-linear perturbation growth over this time period.

**Fig. 5:**
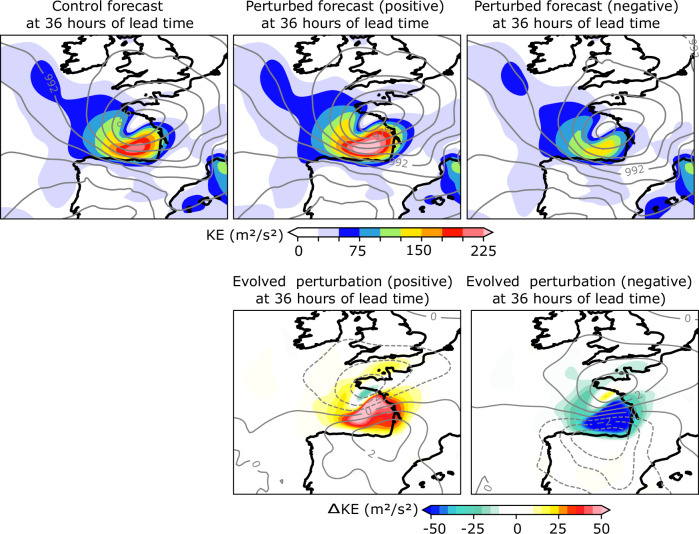
Control and perturbed forecasts, and evolved perturbations of kinetic energy for cyclone Xynthia at 36 h of lead time. First row: Control and perturbed forecasts of kinetic energy (KE) at 36 h of lead time. Contours represent the mean sea level pressure every 4 hPa. Second row: Evolved KE perturbation fields at February 28th 00 UTC. Solid (dashed) lines represent the positive (negative) evolved surface pressure every 2 hPa.

In D14 physics-based simulations driven by adjoint-based perturbations to the initial condition with magnitudes comparable to initial condition uncertainty, were compared to a “control” forecast, suggesting that an even more extreme event than the one that actually happened was plausible (see Figures 6, 12, and 13 in D14). Figure 5 presents the control and perturbed AI forecasts at the 36-h forecast time over the Bay of Biscay, showing the predicted values of kinetic energy (in colors) and mean sea level pressure every 4 hPa (in contours). The forecast shows a low-level jet along the southern area of the low pressure, from the west coast of Spain to the west coast of France. Positive and negative perturbed forecasts increase and decrease the kinetic energy in the region, reaching maxima of 70 and −70 m^2^/s^2^ respectively, while also modifying the low-pressure system, showing a comparable response to the one shown in D14, and also presenting similar spatial structures (Fig. 12 in D14). Differences in the mean sea level pressure between the perturbed and control forecasts are represented by contours in Fig. 5 every 1 hPa, showing a north-to-south gradient for the evolved perturbation fields which is consistent with the results from the dynamical model (Fig. 12 in D14). However, the magnitude of the change in pressure is smaller than the one from D14, which showed evolved perturbation fields every 2 hPa.

## Discussion

AI models are examined as initial condition sensitivity tools to explore the links learned between different fields as the forecast evolves. This provides a mechanism to assess the physical realism of the AI models, which is currently under-explored. So far, these models have produced physically consistent responses to simple dynamical tests^35^, but have failed in preserving key atmospheric balances^33^, and presented error-growth patterns from small-amplitude initial condition perturbations that did not reflect the characteristic “butterfly” effect of dynamical models^36^. Here, sensitivities based on the Spherical Fourier Neural Operator (SFNO) AI model are compared to those from a dynamical model, which are used as references of physically plausible links in the atmosphere. The case of the study is cyclone Xynthia, which was an impactful extreme event in Western Europe in 2010, whose sensitivity fields were already examined by means of dynamical and adjoint models^17^.

The SFNO exhibits high sensitivities for the integrated water vapor, which is consistent with the results from D14, and is aligned with other numerous studies where moisture was identified as one key driver for cyclone development^37^. Moreover, SFNO moisture sensitivity maximums are located over an anomalous warm filament of air within an atmospheric river, the same as in D14. Similar spatial structures between the dynamical and the AI model are found for other variables, such as temperature and the meridional wind velocity, and vertical sensitivities are also remarkably similar for relative humidity. Sensitivity-based perturbations simulate increased values of kinetic energy over the Bay of Biscay, similar to what is found in D14. The AI data-driven model was robust to a set of simple physical tests, such as shifting the perturbation fields 20° West, or 20° West and 25° North over areas of low sensitivity, showing little-to-none differences in the forecasts as compared to the control one (not shown). Moreover, the evolved kinetic energy perturbations always followed the path of the low-pressure system, showing no changes elsewhere in the domain. The sensitivities amplify and grow with an increased forecast lead time, and exhibit wave structures, similar to those usually appearing in the fields from adjoint models. In contrast to the dynamical models (in which geopotential is a derived quantity, not a model state variable), SFNO exhibits a strong dependence on the geopotential, partially masking the influence of the remainder of the variables. This might imply a certain lack of consistent inter-variable links in the model, as exemplified by the behavior of the evolved perturbation fields, where the sea level pressure did not show a change as big as in D14 under moisture-based-perturbation simulations. This strong dependence on geopotential has been further confirmed by conducting similar experiments in other cyclones (Typhoon Lupit^23^; and Typhoon Nuri^38^; not shown), and was also identified in other deep learning models for statistical downscaling, where this behavior was attributed to potential co-variabilities within the set of explanatory variables in the model^29,30^.

The aforementioned properties outline the potential of AI data-driven models to learn physical relationships to some degree, and their ability to automatically identify plausible links between atmospheric variables over space and time. While improvements are still required to capture fully consistent and complex physical relationships, these models are already able to capture important relationships between atmospheric variables, and produce accurate forecasts based on them. One interesting conclusion of this study is that obtaining similar results from different modeling approaches -adjoint of a physical model and an AI model- support the robustness and generality of the results. That is, the general sensitivity characteristics are not model/technique dependent (although the specific details do vary with model). This study clearly exemplifies the potential of these tools for sensitivity studies, especially given the rapid computation of the gradients. Moreover, general properties of neural networks and the backpropagation algorithm can enable unprecedented sensitivity studies at longer timescales^15^ (>5 days), since they are not constrained by linear assumptions, as is the case with the adjoint model, and will be explored in future work.

## Methods

### AI data-driven model: Spherical Fourier Neural Operator (SFNO)

The Spherical Fourier Neural Operator (SFNO) is an AI data-driven model designed to forecast the next 6 h of weather at 0.25° of spatial resolution, given the same set of variables at initial time^7^. Forecasts at longer lead times can be produced by using the model outputs as inputs to the next iteration (auto-regression). SFNO is an updated version of Fourcastnet^6^, which built on the Adaptive Fourier Neural Operator (AFNO^39^) to perform the Fast Fourier Transform (FFT) with a Vision Transformer^40^ backbone, therefore taking advantage of the benefits of self-attention to extract meaningful patterns from spatial data. This model configuration pioneered the use of AI data-driven models for weather forecasting, being the first ever to achieve forecast skill on par with the Integrated Forecasting System (IFS) from the European Centre for Medium-Range Weather Forecasts (ECMWF) -later followed by Graphical Neural Networks-based topologies (GraphCast^8^ and AIFS^10^) or other transformer-based ones (FuXi^9^ and Pangu-Weather^41^). SFNO builds on spherical harmonics as opposed to the FFT of AFNO. The (trained) model can be downloaded from the ECMWF AI-models github: https://github.com/ecmwf-lab/ai-models. Once trained, 36-h forecasts for cyclone Xynthia were produced in the order of minutes with a single CPU, though significantly faster times (on the order of seconds) could be achieved using GPUs.

### Computation of initial condition sensitivities with AI data-driven models

To compute the sensitivity of the kinetic energy (KE) over the Bay of Biscay at the final time (00 UTC, February 28th), relative to the input features at the initial time (12 UTC, February 26th), five steps are followed. The process is outlined below:

1. Standardization of input variables: The input variables are first standardized using the training mean and standard deviation. A complete list of input variables is provided in the “Data” section.
2. Model forecasting: The AI data-driven model’s auto-regression mechanism is then unfolded. Specifically, the model is iterated six times to generate a 36-h forecast, where the outputs from each step are used as inputs for the next.
3. De-standardization of prediction: The prediction is de-standardized by applying the training mean and standard deviation, bringing the results back to the original scale of the variables.
4. Kinetic energy calculation: The kinetic energy is calculated by summing the squares of the predicted zonal (*u*) and meridional (*v*) wind components at each grid point within the Bay of Biscay (43-48°N, 6-0°W, highlighted by the magenta area in Fig. 1). The result is then averaged over the total number of grid points (*N*), such that $KE=0.5{\Sigma }_{i}({u}_{i}^{2}+{v}_{i}^{2})/N$.
5. Sensitivity computation: Finally, the gradients—i.e., the partial derivatives of KE with respect to the input features at the initial time-are calculated using the chain rule and the backpropagation algorithm from PyTorch’s automatic differentiation package. The chain rule for sensitivity computation is outlined in Eq. (1). This step is conceptually similar to the gradient calculations used during model training for gradient descent.

The sensitivity fields were computed within minutes on a single CPU, though significantly faster times (on the order of seconds) could be achieved using GPUs.

1 $$\frac{\partial KE}{\partial {\bf{X}}}=\frac{\partial KE}{\partial {\bf{Y}}}\times \frac{\partial {\bf{Y}}}{\partial {F}_{6}}\times \frac{\partial {F}_{6}}{\partial {F}_{5}}\times \ldots \times \frac{\partial {F}_{1}}{\partial {{\bf{X}}}^{{\prime} }}\times \frac{\partial {{\bf{X}}}^{{\prime} }}{\partial {\bf{X}}}$$

Where **X** and ${{\bf{X}}}^{{\prime} }$ are the raw and standardized input features, respectively, *F*_*z*_ the model output at each auto-regressive step *z*, and **Y** is the de-standardized predicted zonal and meridional wind fields over the Bay of Biscay. Gradients relative to the standardized version of the inputs can be easily computed by ignoring the last term in Equation (1).

### Sensitivity-based perturbations

To facilitate comparison, the perturbations to the initial condition (*Δ***X**) are derived using a strategy similar to that in D14 (Eqs. (2), (3)). First, the sensitivity fields are multiplied by the square of the difference between atmospheric features at the final (forecast, **F**) and initial (analysis, **X**) times (see term *w* in Eq. (2)). This scaling approach ensures that the sensitivities are comparable across variables and levels, preventing any bias that could otherwise lead to suboptimal perturbations. Additionally, this scaling reflects the largest forecast differences, grounded in the model’s behavior rather than arbitrary assumptions. Next, the resulting product is adjusted by a scaling factor, *s* (Eq. (3)). The magnitude of the initial perturbations is chosen to align with analysis error estimates, which typically show maximum values of 1 m/s in the wind field and 1 K in the temperature field. These error estimates are consistent with those found in radiosonde and drop sonde observations as well as those represented in data assimilation systems and thus provide a reasonable lower bound for initial condition uncertainty^17^. Based on this criterion, a unique scaling factor of 0.4 is applied to adjust the perturbations for every variable. Once the perturbation fields are computed, they are added to the initial condition and are evolved using the AI data-driven model. To establish the fairest comparison with D14, every variable in SFNO’s input set was perturbed, therefore following the same approach therein. Hence, for each input variable **X**:

2 $$w={({{\bf{F}}}_{{t}_{0}+\Delta t}-{{\bf{X}}}_{{t}_{0}})}^{2}$$

3 $$\Delta {\bf{X}}=sw\frac{\partial KE}{\partial {\bf{X}}}=0.4w\frac{\partial KE}{\partial {\bf{X}}}$$

### Data

Data for the initial condition is taken from ECMWF Reanalysis version 5 (ERA5^28^), a reanalysis dataset at 0.25° of spatial resolution developed by the ECMWF, that provides atmospheric information at a large number of pressure levels and 1-h intervals for the period 1979-present. ERA5 is built by combining data from a global station network and “first guess” forecasts from Numerical Weather Prediction (NWP) models, by means of data assimilation algorithms, and is known to be the most accurate representation of the atmosphere. SFNO was trained on ERA5 using data at 0, 6, 12, and 18 UTC from the period 1979–2015. SFNO presents the following set of 73 variables: surface air temperature; mean sea level pressure; surface pressure; integrated water vapor; surface zonal and meridional wind; zonal and meridional wind at 100 meters; relative humidity, air temperature, zonal and meridional wind, and geopotential, at the following pressure levels—50, 100, 150, 200, 250, 300, 400, 500, 600, 700, 850, 925, and 1000 hPa. The input variables are standardized using the spatially averaged mean and spatially standard deviation from the training data.

## Data Availability

The datasets generated in this study, including model simulations and sensitivity fields, are available through the University of California San Diego Library^42^.

## Code availability

The underlying code for this study is available in a Github repository and can be accessed via this link: https://github.com/CW3E/AI-sensitivity.
